# Toward a treaty on safety and cost-effectiveness of pharmaceuticals and medical devices: enhancing an endangered global public good

**DOI:** 10.1186/1744-8603-2-5

**Published:** 2006-03-28

**Authors:** Thomas Alured Faunce

**Affiliations:** 1 Medical School and College of Law, Australian National University, Canberra ACT Thomas A Faunce LlB(Hons) BMed PhD, Senior Lecturer. Project Director, Globalisation and Health, Centre of Governance of Knowledge and Development, Regulatory Institutions Network, Australia

## Abstract

• Expert evaluations of the safety, efficacy and cost-effectiveness of pharmaceutical and medical devices, prior to marketing approval or reimbursement listing, collectively represent a globally important public good. The scientific processes involved play a major role in protecting the public from product risks such as unintended or adverse events, sub-standard production and unnecessary burdens on individual and governmental healthcare budgets.

• Most States now have an increasing policy interest in this area, though institutional arrangements, particularly in the area of cost-effectiveness analysis of medical devices, are not uniformly advanced and are fragile in the face of opposing multinational industry pressure to recoup investment and maintain profit margins.

• This paper examines the possibility, in this context, of States commencing negotiations toward bilateral trade agreement provisions, and ultimately perhaps a multilateral Treaty, on safety, efficacy and cost-effectiveness analysis of pharmaceuticals and medical devices. Such obligations may robustly facilitate a conceptually interlinked, but endangered, global public good, without compromising the capacity of intellectual property laws to facilitate local product innovations.

## Background: regulating the global medicines and medical devices industries

The global market for "innovative" pharmaceuticals and medical devices has become one of the most significant sectors for government healthcare spending, particularly as higher corporate rents are leveraged from elevated intellectual property standards[1]. Its influence on public policy is set to expand exponentially, as the products involved are innovatively re-shaped by nano and gene technology and priced accordingly[2]. Aging populations and normal profit-seeking behaviour by multinational corporate manufacturers and private insurers, in a regulatory environment with diminished government controls, are also likely to be major factors[3].

"Medicines" may be divided into subcategories depending on whether they are available to the public by physician prescription or over-the-counter pharmacy sales, have synthetic or biologic components, are patented or generic, or are complementary (outside the traditional medical evidence base) in nature[4]. The term "medical device" has been defined in various terms by regulatory agencies, but generally refers to any instrument, apparatus, appliance, or related article that is intended for use in the diagnosis, prevention, monitoring, treatment, or alleviation of disease, or is intended to affect the structure or function of the human anatomy[5].

Efficacy and safety evaluation are now routine initial regulatory hurdles in most nations for any newly created prescription medicine and medical device. Animal studies (particularly for teratogenicity, carcinogenicity and mutagenicity) and then three phase human clinical trial data, are widely used for institutional approval (licensing or registration) of pharmaceuticals and a variety of other sources for post-approval surveillance[6].

As shall be discussed in more detail, nations such as Canada, Australia, New Zealand and the UK, possess institutions that have achieved international recognition for excellence in cost-effectiveness analysis of pharmaceuticals ("CEAP") as a final component of safety and efficacy evaluation ("SE/CEAP")[7]. The literature and institutional arrangements for cost-effectiveness analysis of medical devices ("CEAMD") after safety and efficacy approval ("SE/CEAMD"), is much less developed[8]. This article will discuss some significant recent industry challenges to such processes.

International benchmark organizations for medicines and medical devices safety and efficacy evaluation, such as the US Food and Drug Administration ("FDA") have also recently come under intense public and governmental scrutiny for perceived inadequacies and conflicts of interest[9]. Additional concerns in this area are corporate-lead international harmonisation processes in safety and efficacy evaluation of medical devices, that appear to undermine the precautionary principle by shifting the burden of proof to public authorities post marketing approval[10]. Given that such regulatory processes are under pressure from multinational industry interests, this article explores whether the most efficient public or State response may be to work toward a multilateral treaty in this area.

## The global spread of medical safety, efficacy and cost-effectiveness analysis

Increasing international interest exists in CEAP prior to government reimbursement as a necessary value approval stage after safety and efficacy evaluation[11]. Australia was one of the first nations to embrace this concept, through Pharmaceutical Benefits Scheme ("PBS") guidelines, in the early 1990s[12]. The resultant processes, operating under the aegis of Australia's Pharmaceutical Benefits Advisory Committee ("PBAC"), are now widely regarded as giving Australia world class expertise in the area[13]. They have a major role in implementing the National Medicines Policy ("NMP") 2000, the four central objectives of which are: timely access to the medicines that Australians need, at a cost individuals and the community can afford; medicines meeting appropriate standards of quality, safety and efficacy; quality use of medicines; and maintaining a responsible and viable medicines industry[14]. A major advantage of the Australian system, in that the monopsony buying power of the Federal government can build on CEAP prior to Federal formulary listing to achieve major price reductions from industry[15].

The New Zealand Pharmaceutical Management Agency ("PHARMAC") was originally established under the Health *and Disabilities Services Act *(1993) (NZ) (now the *Public Health and Disability Act 2000 *(NZ)) with the specific purpose of improving the management of Government expenditure on pharmaceuticals already approved on safety and efficacy grounds. PHARMAC, with the assistance of independent medical experts on the Pharmacology and Therapeutics Advisory Committee ("PTAC") and its specialist sub-committees, manages, on cost-effectiveness grounds set out in guidelines, a Federal formulary, known as the Pharmaceutical Schedule. Patients and their advocacy groups have input into PHARMAC's listing decisions through a Consumer Advisory Committee. One of its major advances involves the use of tendering for low cost generic medicines[16].

Cost-effectiveness evaluation was introduced as a interrelated evaluation with safety and efficacy approval, by the Canadian provinces of Ontario[17]. and British Columbia in the early 1990's[18]. The Canadian Expert Drug Advisory Committee ("CEDAC") now operates under the Coordinating Office for Health Technology Assessment ("CCOHTA") to create cost-effectiveness recommendations for ten provincial and three territory governments, as well as specific Federal programs (for example, veterans and also indigenous people)[19]. The Canadian Patented Medicines Prices Review Board ("PMPRB") sets a maximum "factory gate" price for new, patented "breakthrough" drugs, based on the median price in seven OECD nations specified in regulations (France, Germany, Italy, Sweden, Switzerland, U.K. and the U.S.), most of which (apart from the US) rely on some form of CEAP to guide government reimbursement decisions. The PMPRB attempts to also ensure that most new patented drug prices are limited to those of comparable pharmaceuticals sold in Canada and that existing patented drug prices in that nation cannot increase by more than the Consumer Price Index (CPI), or become the highest in the world[20]. Although it does not advertise the fact, the PMPRB appears to utilise a form of CEAP[21].

In Europe safety and efficacy considerations fall within the European Medicines Agency Guidelines on Risk Management Systems for Medicinal Products for Human Use[22]. Governments in most OECD countries (as well as those mentioned above) utilise forms of CEAP in conjunction with safety and efficacy evaluations[23]. Belgium, Finland, Norway, Portugal and Sweden have introduced formal cost-effectiveness as a routine "fourth hurdle" after quality, safety and efficacy determination[24]. The Hungarian Office of Health Technology Assessment of the National Institute for Strategic Health Research has a mandatory role in granting social insurance subsidies related to medicines and medical devices[25]. The resultant expert recommendation may allow the creation of formularies for either positive or negative government reimbursement of pharmaceutical prices[26]. As well as cost-effectiveness, cost-utility, cost-benefit and cost-minimisation approaches are utilised[27]. CEAP is often linked with reference pricing, which may involve a government reimbursing the average or lowest price in a therapeutic grouping of prescription medicines[28]. The UK Pharmaceutical Price Regulation Scheme ("PPRS")[29]. links government control over manufacturer profits with a negative (non-reimbursed) list and cost-effectiveness guidance from the National Institute of Clinical Excellence ("NICE")[30]. Though also utilising expert analysis of systematic reviews and modelling, unlike Australia's PBAC, NICE commissions evaluations on classes of drugs, rather than having them performed by submitting corporations for specific products[31].

In the United States, safety and efficacy evaluations follow the FDA pharmacovigilance and risk management action plans[32]. CEAP is less widely utilised in conjunction with safety and efficacy analysis at the Federal level[33]. The same true in Japan[34].Industry critics have pointed to methodological flaws such as vague definitions of therapeutic class and the difficulty of obtaining good measures for societal preferences[35]. Politically dominant private insurance and pharmaceutical corporations have also linked CEAP with claims that indiscriminate, non-evidence-based government charges could impede patient choice concerning "innovative" medicines[36]. Individual healthcare facilities (with limited bargaining power) in the US are encouraged by industry to develop formularies useful to patient care using managed care guidelines[37]. A group of States have organised a Drug Evaluation Review Process ("DERP") to assist their managed care plans[38]. Health Management Organisations ("HMO's") have begun to require pharmaceutical manufacturers to make formulary submissions according to guidelines prepared by the Academy of Managed Care Pharmacy ("AMCP") and increased prominence has been given to the work of the Agency for Health Research and Quality ("AHRQ")[39]. Increasing prominence has also been given to CEAP performed by the Veterans Health Administration ("VHA") and the Pharmacoeconomics Evaluation Center ("PEC") of the Department of Defence[40].

CEAP and CEAMD are emerging fields of academic and health policy interest for China, with the particular aim of reducing the high proportion (44%) of pharmaceutical expenditure in total healthcare expenditure[41]. The South Korean government has been developing pharmaco-economic guidelines after consultations with experts in Canada and Australia[42]. In 2001 the Singapore Ministry of Health appointed a Drug Cost Review Task Force to revise cost-effectiveness processes in connection with a Standard Drug List[43]. In Thailand, three taxation funded public insurance schemes provide a minimum pharmaceutical package through a cost-effectiveness evaluated National List of Essential Drugs[44]. Malaysia and Pakistan have governments very interested in cost-effectiveness analysis of pharmaceuticals, but evaluations are limited by lack of funding, lack of trained personnel, lack of protected research time, limited access to data and information, poor dissemination and official uptake of research outcomes[45].

Developing countries in general frequently lack the resources to train and support officials with the requisite pharmaco-economic expertise to permit interlinked safety, efficacy and CEAP/CEAMD evaluations on an effective, national scale[46]. To respond to community (and their own employees') social justice concerns about public health problems arising from high intellectual property rents, the multinational pharmaceutical industry has proposed self-regulatory alternatives emphasising pharmaco-philanthropy, public-private partnership initiatives and covert differential pricing[47]. Many developing nations, such as India, rely upon the World Health Organisation's ("WHO") Essential Medicines List[48]. This assesses cost of such pharmaceuticals per case, per cure, per month of treatment, per case prevented, per clinical event prevented, or, if possible and relevant, cost per quality-adjusted life year gained[49].

The intense recent interest focused on the global problems with safety and cost-effectiveness of pharmaceuticals, has lead to medical devices becoming somewhat of a silent partner in such regulatory discussions. The International Society for Pharmacoeconomics and Outcomes Research ("ISPOR") is attempting to redress this imbalance[50]. Devices do create unique difficulties, particularly through difficulties obtaining blinded trial data, the skill involvement with diagnosis (they are not therefore fully embodied technologies and have cost-effectiveness learning curves), the frequency of product modifications and poor development of regulatory theory in this area[51]. The Global Harmonization Task Force (GHTF) comprises representatives from national medical device regulatory authorities and industry from European Union, the United States of America, Canada and Japan was established ostensibly to encourage convergence in safety, efficacy and cost-effectiveness evaluations, whilst also promoting technological innovation and facilitating international trade[52].

An important point to note from the above survey is that established and effective forms of CEAP and CEAMD work in close conceptual association with safety and efficacy evaluations. We can now examine whether it may make better regulatory sense to consider these as integrally linked processes.

## Advantages and disadvantages of SE/CEAP and SE/CEAMD

Affordable access to essential medicines is increasingly recognised as a global public good, providing an essential precondition to a reasonable quality of life for a significant proportion of every human population, being systematically underprovided by private market forces and imposing burdensome international externality costs on third parties[53]. Further, affordable access to essential medicines appears to be emerging, both academically and in practise, as a core part of the international right to health in article 12 of the *International Covenant on Economic, Cultural and Social Rights *(article 25 of the *Universal Declaration of Human Rights)*[54]. One recent manifestation was the *Doha Declaration*, which affirmed the capacity of WTO members to use to the full exceptions in the Trade Related Intellectual Property Rights agreement ("TRIPS") to promote public health by facilitating access to affordable medicines[55]. It is also specifically referred to in article 14 of the UNESCO *Universal Declaration on Bioethics and Human Rights*[56]. There seems to be little reason why in theory or practice, affordable access to essential medical devices should not to subject to the same considerations.

SE/CEAP and SE/CEAMD processes, however, despite their value to contemporary health technology assessment and their capacity to facilitate access to medicines, have not themselves been widely discussed as a global public good, or as in any obvious way connected with normative systems of distributive justice and the international human right to health. Neither is primarily regarded as a cost-containment strategy, chiefly because their related formularies generally lack a capped budget and their fiscal effects are predicated on prescribers adhering to recommended indications[57]. SE/CEAP and SE/CEAMD, create no barriers to market access, or infringements of intellectual property rights. They merely attempt to rationalise, according to scientific evaluation of a hierarchy of clinical trial evidence, government or other third party (private health insurer) reimbursement expenditure [58].

SE/CEAP and SE/CEAMD have three key advantages, which may allow them to evolve into an important global public good. The first involves an emphasis on scientific evidence, the second a commitment to equity, to ensuring value for a whole community and the third, the capacity of SE/CEAP and SE/CEAMD to act as fiscal brakes on rent flowing to prior intellectual property owners without inhibiting encouragement of local innovation through high intellectual property protection.

One of the major disadvantages of SE/CEAP and SE/CEAMD, is the common presence of methodological flaws either in the evaluations by regulators, or in economic submissions made by industry[59]. SE/CEAMD faces comparative difficulties with "blinding," variable physician technique and a shorter product life cycle. Yet, they may benefit from easier *in vitro *assessment and a greater capacity to characterise incremental design changes by laboratory bench testing.

Another disadvantage, from the regulators' point of view, is the lack of "hard" outcome data such as Quality Adjusted Life Years ("QALYs"), particularly at initial evaluation of an innovative product. Manufacturers often claim it is too early to produce such published trial data and prefer to rely on surrogate outcomes, such as readily measured changes in biochemical markers of disease. Another disadvantage is that CEAP and CEAMD analysis is often (unless it is linked to Federal monopsony buying power) unable to question the initial price given by industry. Direct, rather than inferred, evidence of marginal cost of production is denied to evaluators, often on "commercial-in-confidence" grounds. This means that CEAP and CEAMD however excellently performed, often metaphorically take place on an uncertain foundation[60]. There is also issue of nations training enough pharmaco-economic experts to facilitate CEAP and CEAMD for, for example, both pre and post reimbursement listing.

SE/CEAP and SE/CEAMD also commonly be "gamed" by industry. If, for example, in a system such as that of Australia, if a safety regulator approves 5 clinical indications, this could lead to submissions to a cost-effectiveness evaluator on only one indication with the industry expectation of prescription "leakage" outside recommendations, compromising fiscal savings for the taxpayer. Similarly, expert evaluations considering a medicine's toxicity may play an important CEAP role by factoring disutility into modelled analysis, calculating compliance, or altering indications.

Hasty safety approvals could endanger public health, yet heightened industry pressure for "fast-tracking" may arise from diverse sources: prior notification of submission schemes, differing standards of proof, industry applications "salami slicing" indications to fit "orphan" drug categories, by inadequate conflict of interest protections given full cost recovery from industry and pressure for development collaborations with regulators. Over-cautious rejections could delay patient benefits, reduce export earnings and stifle investor confidence; yet safety classifications of innovative nanotechnology products at the device/medicine 'boundary' will be distinctly complex. The public may react adversely to new internationally harmonised medical devices safety regulations that shift burdens of proof to safety regulators after approval, possibly in anticipation of the difficulty in obtaining credible published trial data in this area (recruitment of subjects to nanomedicine safety and cost-effectiveness trials will be unusually difficult). The limited published systematic reviews, may unduly restrict SE/CEAP and SE/CEAMD for nanotechnology products to surrogate outcome measures, rather than quality-adjusted life years.

## Threats from global industry interests

Though well entrenched in the policies of most States, evolution and enhancement of SE/CEAP and SE/CEAMD as a global public good should not be taken for granted. Brand name pharmaceutical multinationals, in particular, are currently involved in a global strategy, using international trade arrangements, carefully funded and seeded academic articles, strategic surveys of relevant processes in Europe and Asia (and how well they respond to the corporate lobbying principle of innovation), to separate cost-effectiveness analysis from safety and efficacy evaluations and central government monopsony buying power and replace it with medicines provision models emphasising privatised insurance,[61]. medicines savings accounts[62]. and direct-to-consumer advertising[63]. This process has already produced large scale adverse public health consequences in China[64]. and Singapore[65]. Nevertheless it is still being promoted by industry as a credible policy alternative to universal taxpayer-funded access schemes in developed nations such as Australia, usually in the guise of enhancing "consumer" choice and responsibility[66]. Critics point to the lack of logic or compassion in industry emphasising the decision-making capacity of sick people, particularly the disabled and poor patients, concerning their health and therapies, as if what they were purchasing was a new car, house, or suit of clothes.

The United Nations Human Development Report 2005 has emphasised, for example, that the World Trade Organisation's ("WTO's") corporate-sponsored Trade-Related Intellectual Property Rights (TRIPS) agreement, along with so-called "TRIPS-Plus" intellectual property protections in subsequent bilateral trade agreements, pose a "pronounced" threat to global public health, particularly through their expansive effect on prices for so-called "innovative" medicines[67]. The US pharmaceutical industry also has a powerful influence on the globally influential US legislature[68]. The *Medicare Prescription Drug Improvement and Modernization Act *2003 (US), as one instance, thwarted attempts to introduce a Federal PBAC-type process in the US, specifically prohibiting the US government from using its bulk buying power for Medicare beneficiaries from negotiating medicines price discounts in a PBAC-style approach[69]. A Congressional Conference Agreement on this legislation obligated US negotiators on the AUSFTA to report on whether that deal offered opportunities to achieve the objectives of the *Bipartisan Trade Authority Act 2002 *(US) including the "elimination of government measures such as price controls and reference pricing which deny full market access" for US pharmaceuticals[70].

Section 1123 of the *Medicare Prescription Drug Improvement and Modernization Act *2003 (US), commissioned a study by the US Department of Commerce, on so-called pharmaceutical "price controls" implemented by SE/CEAP systems in thirteen OECD countries. It claimed that these cost US drug purchasers from $5–$6 billion per year. It argued that US drug prices should serve as a benchmark for deregulated prices, despite the fact that they are 18–67% higher than those in the relevant OECD countries[71].

An important issue here may be the role of Article 64 of the *Agreement on Trade-Related Aspects of Intellectual Property Rights *("TRIPS")[72]. The United States, for example, subsequently has argued that the initial and subsequent moratoria is over and the Non-Violation-Nullification of Benefits ("NVNB") remedy must now be accepted, by all WTO Members, as applying to the TRIPS Agreement[73]. At the WTO meeting in Hong Kong in December 2005, United States negotiators attempted to obtain concessions in return for their support for the continuance of the NVNB moratorium[74]. NVNB claims, permitting dispute resolution proceedings for breaching the "spirit" of a trade agreement could both support and undermine CEAP, depending on the undertakings made about it at the time such agreements were entered. The Australian government, for example, quite explicitly gave undertakings that the fundamental architecture of Australia's CEAP system would not be altered by the AUSFTA[75] and backed this up by passing implementing legislation against the process of patent "evergreening" predicated on such an assumption. Crucially important in this context could be Annex 2C (1) of the Australia-United States Free Trade Agreement ("AUSFTA,") where "innovation" is uniquely linked with the socially-oriented concepts of 'high quality health care', 'affordability', 'accountability' and "objectively demonstrated therapeutic significance'. Whether "innovation" should sit within CEAP, or the patent system, or both, is a major conceptual conundrum that probably goes to the heart of the industry agenda in this area.

On 1–2 December 2005, a meeting took place in Paris under the auspices of the OECD "Project on Pharmaceutical Pricing Policies and Innovation." Inclusion of the term "innovation" in the title discloses what was probably the chief purpose of this Project (though attempts were made by the US delegation to obfuscate this agenda, particularly by initial statements ostensibly withdrawing support and ensuring a significant role for nations such as Canada and Mexico). This was to broach the first stages of implementation of the US Department of Commerce report mentioned previously. Its stated terms of reference appear to confirm this. They are:

1) to add to the base of information about pharmaceutical pricing policy in OECD countries and develop a taxonomy and framework for making international comparisons of policies [the European Union was running a similar investigation already]

2) to analyze cross-national impacts and implications of policies, particularly with respect to impact on pharmaceutical prices paid in other countries and impact on pharmaceutical research and development[76].

## Toward a multilateral treaty

It seems remarkable, in an age of corporate globalisation, that medicines and medical devices national safety regulators and cost-effectiveness evaluators continue to work largely in formal isolation to assess the same products. Given the importance of SE/CEAP and SE/CEAMD to sustainability and legitimacy of public health systems, it is also peculiar that governments have not already perceived the advantages of creating a multilateral treaty in this area.

One intermediate suggestion is to include provisions establishing SE/CEAP and SE/CEAMD committees or working groups in bilateral trade agreements. The aims of such arrangements would include fostering relevant international regulatory collaborations, capacity building expertise (by facilitating the relevant trade in services) and overcoming regulatory safety concerns that might provide barriers to the entry of cheap generic medicines (for example from China to Australia). Such provisions would not impact adversely on intellectual property rights. Consequently, they would not infringe any prohibitions on restricting intellectual property rights or discriminating against fields of technology emerging from the TRIPS Agreement.

For each such provision, a government department (usually the respective Ministries of Health) would need to assume responsibility for operationalising the related obligations and requirements. Qualifications and process of appointment of relevant experts would need to be resolved, as would the reporting mechanisms. Establishing such a mechanism in a trade agreement would promote SE/CEAP and SE/CEAMD expertise in relevant universities, building careers in this area, with the prospects of governments saving more money as greater numbers of relevant experts become available to preform both pre and post-listing evaluations.

Such a provision might be as brief as the following annex at the end of a trade in goods chapter:

"**Medicines and Medical Devices Safety, Efficacy and Cost-Effectiveness Committee **The Parties hereby establish this Committee, comprising relevant officials and expert advisors from each Party. Its primary objective shall be to promote discussion and mutual understanding, collaborations, training, education and sharing of expertise with a view to enhancing and developing techniques of, and research related to, safety, efficacy and cost-effectiveness evaluations of medicines and medical devices."

In time, the increased interest in SE/CEAP and SE/CEAMD generated by such provisions may lead to a *Treaty on Safety, Efficacy and Cost-Effectiveness Evaluation of Medicines and Medical Devices*. Such a Treaty could be sponsored either by UNESCO, or the World Health Organisation ("WHO") or, hopefully, both organizations in collaboration.

The relevant terms of reference could involve negotiations in the following areas:

1) the appropriate interrelationship of safety, efficacy and cost-effectiveness evaluations

2) the social theories that should underpin such evaluations including the blance between global public goods and private rights, perspectives on the relative importance and interaction in this context of bioethical equity and social justice, the international human rights to health, international trade norms preventing non-tarriff barriers and industry lobbying principles such as recognition of innovation.

3) how to improve access by regulators, health professionals, consumers and industry to public data bases of large-scale, randomised, double blind clinical trails involving head to head comparisons using therapeutically equivalent dosage forms for the most commonly prescribed pharmacological analogues or non-drug therapies for the same indication.

4) whether SE/CEAP and SE/CEAMD can progressively involve greater use of "hard" outcome measures, such as deaths prevented or quality-adjusted life years (QALYs) gained, rather than "surrogate" pharmacological outcomes (for example low density lipoprotein levels or blood pressure).

5) improving existing SE/CEAP and SE/CEAMD systems efficiencies in specifics such as reference pricing and tendering for ultra low cost generic medicines, but also whether the concept of "innovation" in relation to medicines and medical devices should be defined to include elements of safety, efficacy, affordability and objectively demonstrated therapeutic significance.

6) discussions on post marketing responsibilities which could include price-volume and binding health outcome agreements between regulators and industry, as well as the appropriate structure of vigilance trials, adverse incident reporting, impact of fraud, prescribing habits and alternative or complementary therapies.

7) discussions on how to globally capacity build SE/CEAP and SE/CEAMD as a career for health professionals and facilitate trade in services training programmes, expert exchanges and collaborations.

8) discussions on improving data in areas such as choice of comparitor, measurement of relevant costs and benefits, length of follow up, peculiarities of local setting and appropriate valuation of economic, clinical and patient-reported (or humanistic) outcomes.

9) negotiations on public interest limits about commercial-in-confidence protections and on disclosing local and international marginal costs of production for each drug. Important principles on the issue of commercial-in-confidence, for example, emerging from the parallel processes of UK NICE and Canadian CCOHTA, are that it should not so inhibit transparency as to prevent manufacturers disclosing enough information to make their submission understandable to the public or governments, or that it should not endanger public safety and should not be set unilaterally by industry[77].

10) horizon scanning processes to ensure all Parties are speedily appraised of recommended SE/CEAP and SE/CEAMD regulatory responses to developments in new fields such as nano and gene-based technologies.

## Conclusion

This article has argued that despite its obvious attraction to fiscally responsible governments in a time of ageing demographics, neither the continuance, nor enhancement of science-based SE/CEAP and SE/CEAMD processes should be taken for granted. Nation states are just becoming used to the change in sovereignty associated with fully privatised healthcare sectors coexisting with international trade obligations to provide national treatment to multinational corporations. In this context, much official concern has been expressed about growing public disenchantment with the policy influence of the multinational pharmaceutical industry[78].

There are both responsive and pro-active reasons for seeking to include provisions facilitating SE/CEAP and SE/CEAMD in bilateral and multilateral trade agreements. The responsive reason relates to ensuring a more transparent debate about the future enhancement of these processes in relation to an industry agenda which often appears to perceive their stringent application as an impediment to their freedom to manufacture, obtain speedy safety and efficacy approval and market direct to both patients and physicians, with only limited stringent scientific scrutiny about either the marginal cost of production or overall comparative worth to the community.

The pro-active reasons for including SE/CEAP and SE/CEAMD in trade agreements relate to the possibility of creating an important, transparent playing field where the next generation of great debates between public goods and private rights in this sector can take place. They also concern the facilitation of trade-in-services, capacity building relevant expertise, improving relevant processes (including the efficiency of sharing data and reviews), as well as the need to commence negotiations with pharmaceutical multinationals on a more rational approach to important issues such as commercial-in-confidence and marginal cost of production.

Possible disadvantages in proceeding this way include the possibility of such a treaty becoming a lightning rod for a contrary agenda by the pharmaceutical and medical device industries. The aims of such a treaty, for example, could be altered to provide a vehicle for corporate strategies such as "linkage" of regulatory evaluation of a generic pharmaceuticals patent status with quality and safety evaluation prior to marketing approval, or reversal of the precautionary principle with regard to regulatory approval of new medical device technologies.

At this point in the age of corporate globalisation, perhaps it is time to start respecting scientific cost-effectiveness evaluation of medicines and medical devices as a potentially endangered global public good, which should not be conceptually or operationally separated from safety and efficacy evaluations. Governments wishing to take a popular strategy to elections with an ageing population could promote the type of multilateral treaty discussed here (or provisions facilitating SE/CEAP and SE/CEAMD in bilateral trade deals) as a rational and scientific way of restraining medicines prices and ensuring value for public expenditure in this area of the health sector.
